# Supplementary material for: Holstein Friesian dairy cattle edited for diluted coat color as a potential adaptation to climate change
Source: BMC Genomics. 2021 Nov 26;22:856. doi: 10.1186/s12864-021-08175-z (PMC8626976; doi:10.1186/s12864-021-08175-z)
Supplement: Supplementary file 1 — Additional file 1: Table S1. PCR primer, probe, gRNA and repair template sequences used to characterise the PMEL locus and white spotting genes. Table S2. Genotype for major-effect QTL associated with white spotting. Fig. S1. Target site sequence of the genome edited calves. Fig. S2. The edited claves are homozygous for the MC1R ED allele. Fig. S3. Absence of a plasmid-specific fragment in genomic DNA from edited calves. Fig. S4. Original gel photo used to generate Fig. S3. Fig. S5. Absence of mutations at the top three predicted off-target sites. Fig. S6. Absence of characteristic hair phenotypes associated with rat tail syndrome. [file 12864_2021_8175_MOESM1_ESM.pdf]

Supplementary material

Holstein Friesian dairy cattle edited for diluted coat color as a potential adaptation to climate change

Götz Laible, Sally Cole, Brigid Brophy, Jingwei Wei, Shane Leath, Swati Jivanji, Mathew D. Littlejohn and David N. Wells

Table S1. PCR primer, probe, gRNA and repair template sequences used to characterise the *PMEL* locus and white spotting genes.

Name	Sequence	Application
122_F	GGCTCTGATGGGTGTTCTTC	gRNA, *PMEL*
129_F	ATGGGTGTTCTTCTGGCTGT	gRNA, *PMEL*
130_F	TGGGTGTTCTTCTGGCTGTA	gRNA, *PMEL*
ssODN	GGAGAGAAAAACCAGAGCAGGTGTGCAACCCCAAATTCACAC TTGTTCATGTCCAACATCCCACACTCACCTTCTGTGGTCCCTAC AGCCAGAACACCCATCAGAGCCACATGGAGAAGGTATTTTC	HDR template, *PMEL*
1249	TGCTTTAAGATGAGACTGACC	Mutation- specific PCR, *PMEL*
1305	AGCCAGAACACCCATCAG	Mutation- specific PCR, *PMEL*
1283	TTGCTGGAAGGAAGAACAGG	PCR/ddPCR primer, *PMEL*
1284	GGAGACACCTGAAGCACTAC	PCR/ddPCR primer, *PMEL*
1287	TGGGTGTTCTTCTGGCTGTAGGGACCACA	Drop-off probe (HEX), *PMEL*
1285	TGATGGGTGTTCTGGCTGTAGGGACCACA	HDR probe (FAM), *PMEL*
1289	GGCTCTGATGGGTGTTCTTCTGGCTGTAGGGACCACAG	Dark probe, *PMEL*
1286	TGCACACCTGCTCTGGTTTTTCTCTCCCT	Reference probe (FAM), *PMEL*
1670	CCAGCCACCCTCCCCTTCACC	PCR, *MC1R*

1671	CGCAATGATCCTCCACGCTCG	PCR, *MC1R*
1041	ACTATCATATGCTTACCGTAAC	PCR, gRNA/Cas9 plasmid
795	GGGCCATTTACCGTCATTGA	PCR, gRNA/Cas9 plasmid
211	TGCCCCAGAGAAGAGAAGG	PCR, *LALBA*
212	ATTGCTAACGGGAGTGAAGTAAGT	PCR, *LALBA*
1664	GTGATTTGGGTCCCTCTGGG	PCR, OFF 1
1665	GCTGTGCCTAAGGTCCCAAT	PCR, OFF 1
1666	GCACGACTGAGGGACTTTCA	PCR, OFF 2
1667	AACTCATCTCCCGCTACCCT	PCR, OFF 2
1668	GGCCTTAGGGAGCAGACTTG	PCR, OFF 3
1669	TGGAATGTGTGGGCTCCATC	PCR, OFF 3
KIT_F	TGGTGAAGGAGGCATGTCTG	PCR, *KIT*
KIT_R	GGTGTGCCTTTGTGAATTCA	PCR, *KIT*
MITF_F	CGAGACACCACCGGAAACTT	PCR, *MITF*
MITF_R	TTCTGTGTTTGGAAGGGGCC	PCR, *MITF*
PAX3_F	ATGTTAGGTGCAGGTGGAGC	PCR, *PAX3*

PAX3_R	GCTTCCCACCTTGACCTCTC	PCR, *PAX3*

Table S2. Genotype for major-effect QTL associated with white spotting.

QTL*	ID	Position ARS_UCD1.2	Q allele+	q allele#	BEF2 gen@	CC14 gen&
chr2_PAX3	rs109979909	chr2:110817975	A	C	AC	AC
chr6_KIT	rs451683615	chr6:62557125	A	G	AA	AA
chr22_MITF	rs209784468	chr22:31651379	A	G	AA	AA

* QTL from Jivanji et al. 2019; positions reference tag variant or candidate mutation

+ Allele associated with increased spotting

Allele associated with decreased spotting

@ parental cell line

& edited cell line

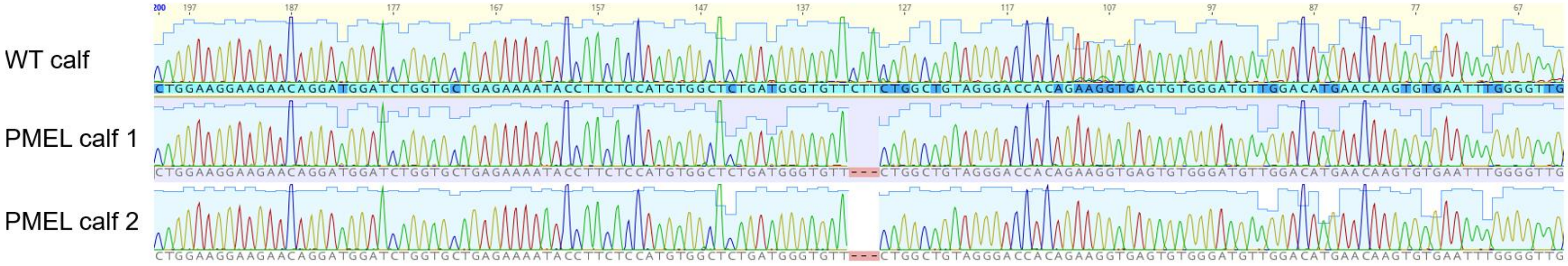

Fig. S1. Target site sequence of the genome edited calves. Shown is an alignment of Sanger sequence results of the *PMEL* target region of one wt calf and the two mutant calves, genome edited for the p.Leu18del PMEL mutation.

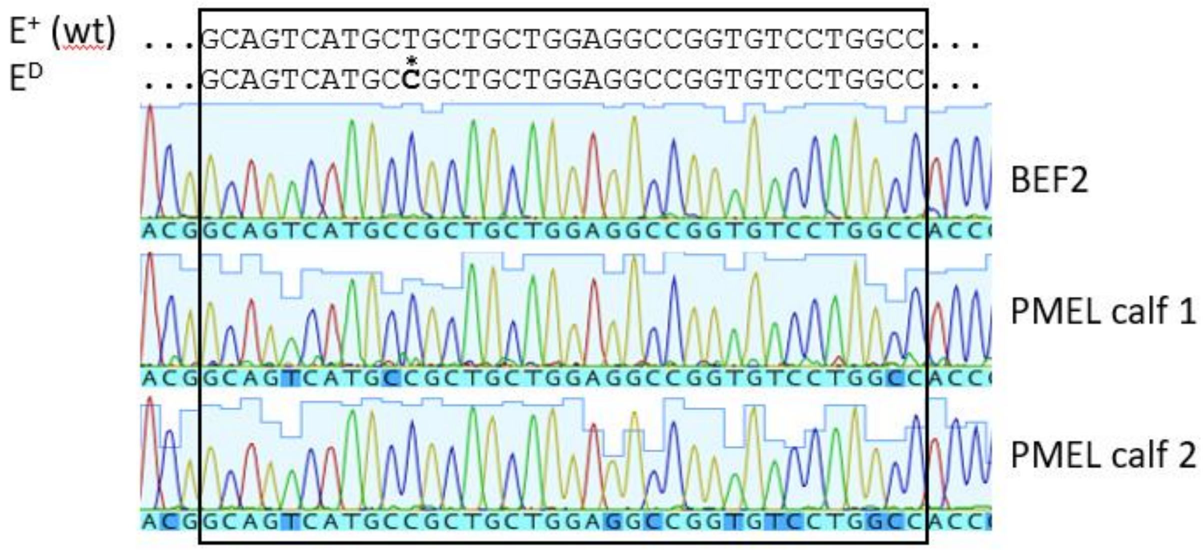

Fig. S2. **The edited claves are homozygous for the *MC1R E*$^{\mathbf{D}}$ allele**. Shown are the Sanger sequence results for the *MC1R* region covering the causative sequence variant of the *E*^{D} allele for the non-edited parental cell line BEF2 and the surviving calf (PMEL calf 1). For comparison, the sequence variation (bold, asterisk) between the E+ (wt) and E^{D} *MC1R* alleles is depicted at the top.

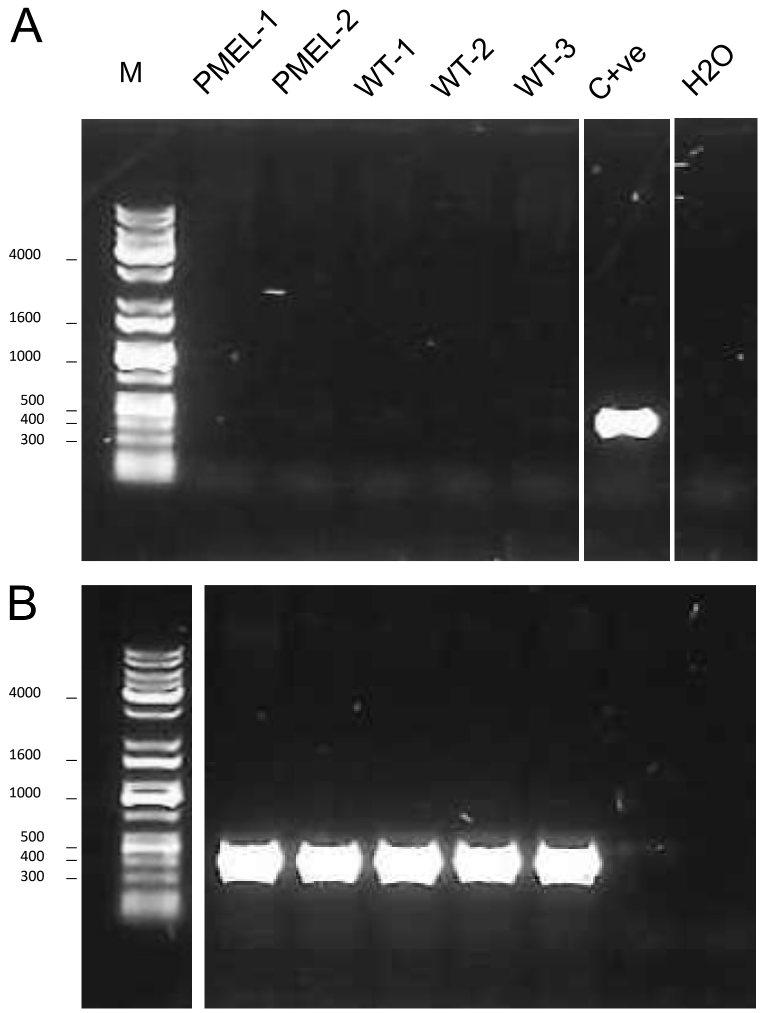

Fig. S3. Absence of a plasmid-specific fragment in genomic DNA from edited calves. (A) Shown are amplification results for a gRNA/Cas9 plasmid-specific amplicon with genomic DNA isolated from the two edited calves (PMEL-1, 2) and the three non-edited control calves (WT-1, 2, 3). M: DNA size marker; C+ve: positive control of gRNA/Cas9 plasmid; H_2O: water control. (B) The same samples analysed for the amplification of a genomic fragment specific for the endogenous *LALBA* gene encoding alpha-lactalbumin. Please note, the pictures in panels A and B have been cropped and relevant lanes were assembled from the original gel photo (shown in Fig. S4) for clarity.

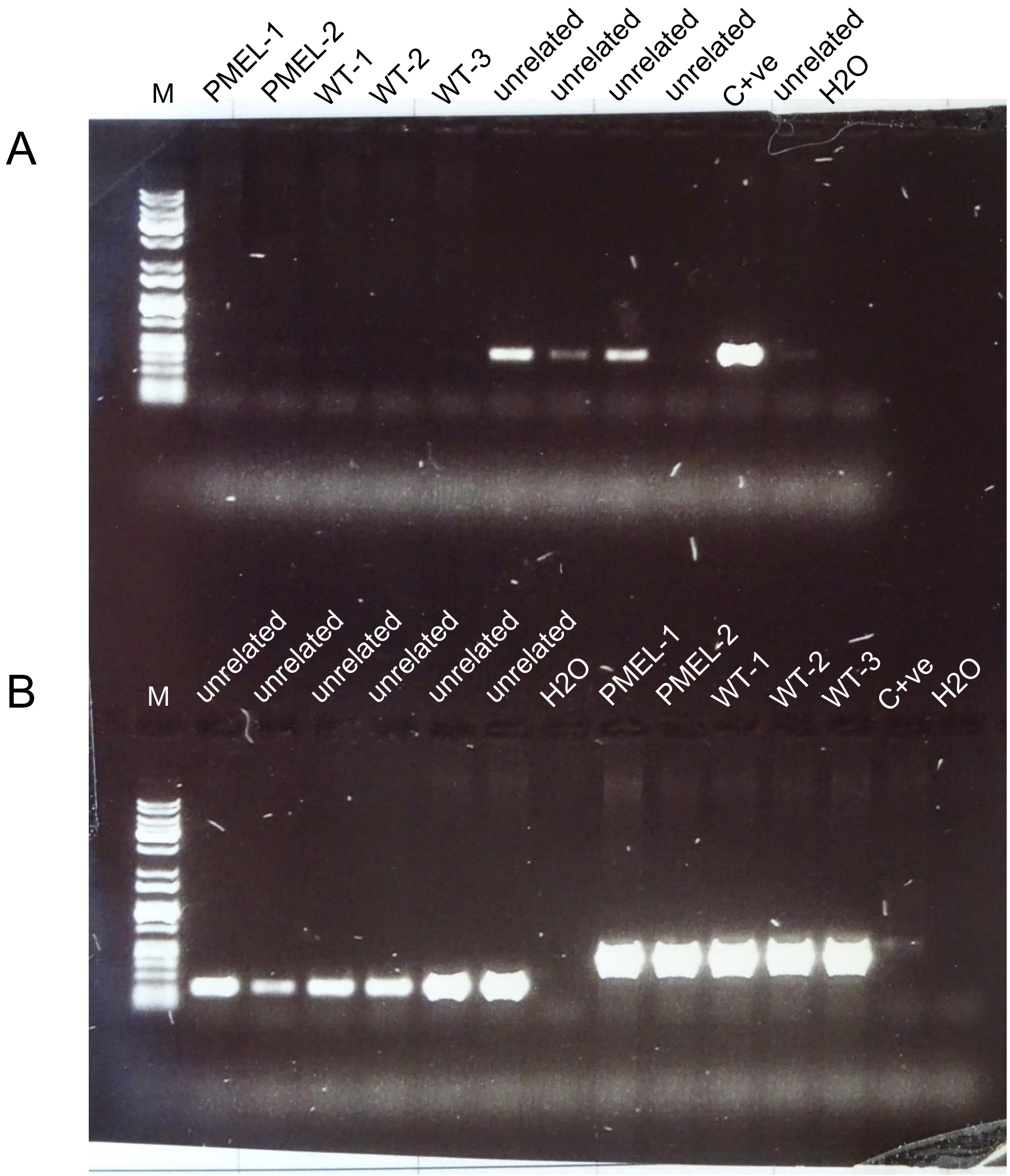

Fig. S4. Original gel photo used to generate Fig. S3. (A) Shown are amplification results for a gRNA/Cas9 plasmid-specific amplicon with genomic DNA isolated from the two edited calves (PMEL-1, 2) and the three non-edited control calves (WT-1, 2, 3). M: DNA size marker; C+ve: positive control of gRNA/Cas9 plasmid; H_2O: water control. Lanes labelled as 'unrelated' show PCR results of an unrelated project in a different species. (B) The same specified samples as indicated above (PMEL, WT, C+ve), analysed for the amplification of a genomic fragment specific for the endogenous bovine *LALBA* gene encoding alpha-lactalbumin. Unrelated: PCR results of an unrelated project in a different species.

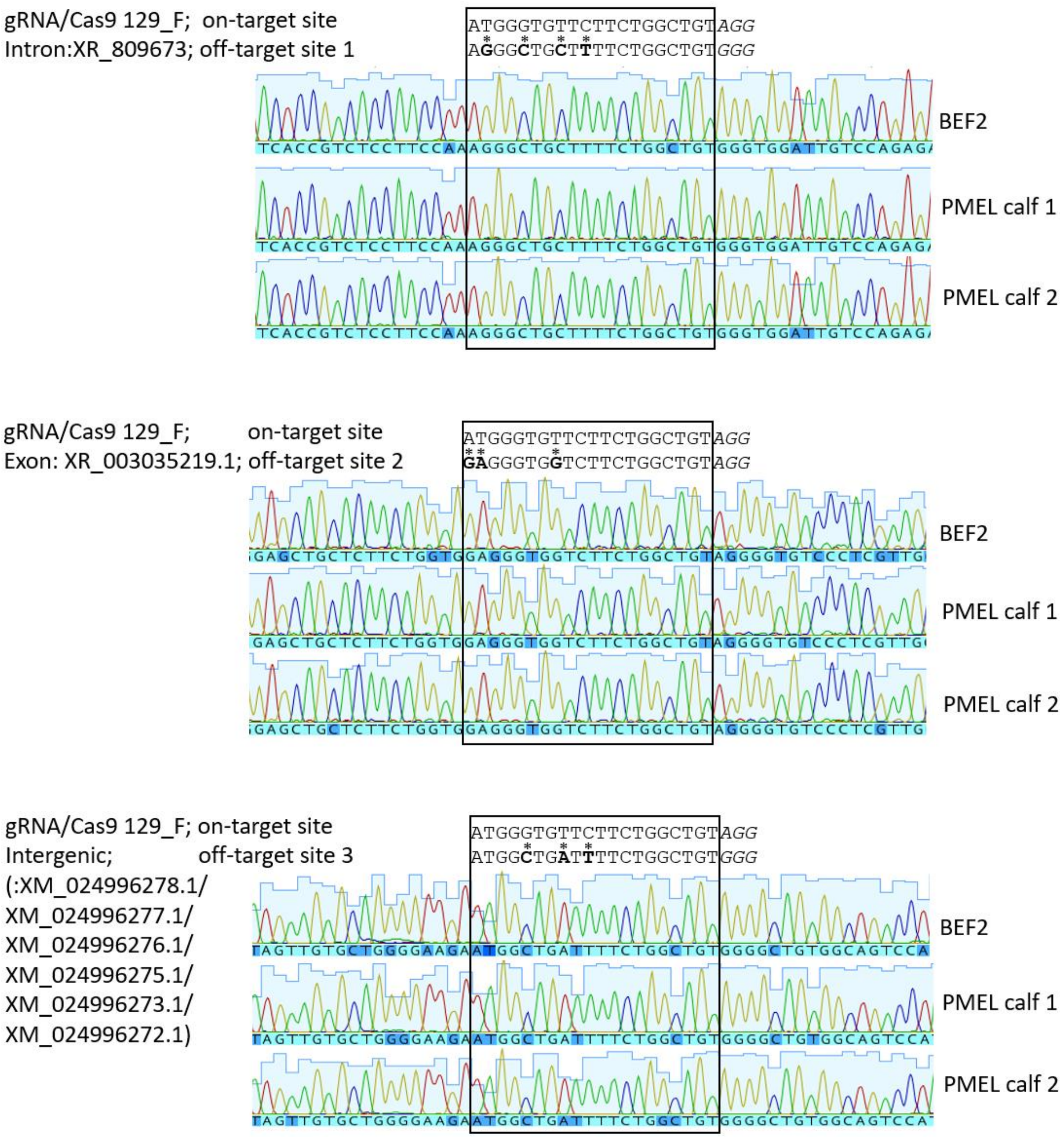

Fig. S5. Absence of mutations at the top three predicted off-target sites. An alignment of on-target sequence and each of the three off-target sites is given at the top. Differences to the on-target sequence are highlighted in bold and an asterisk. The corresponding PAM sequence is shown in italic. Below are the Sanger sequence results for the three predicted off-target sites for the non-edited parental cell line BEF2 and the two edited calves. The box indicates the potential Cas9/gRNA 129F binding site at the off-target site.

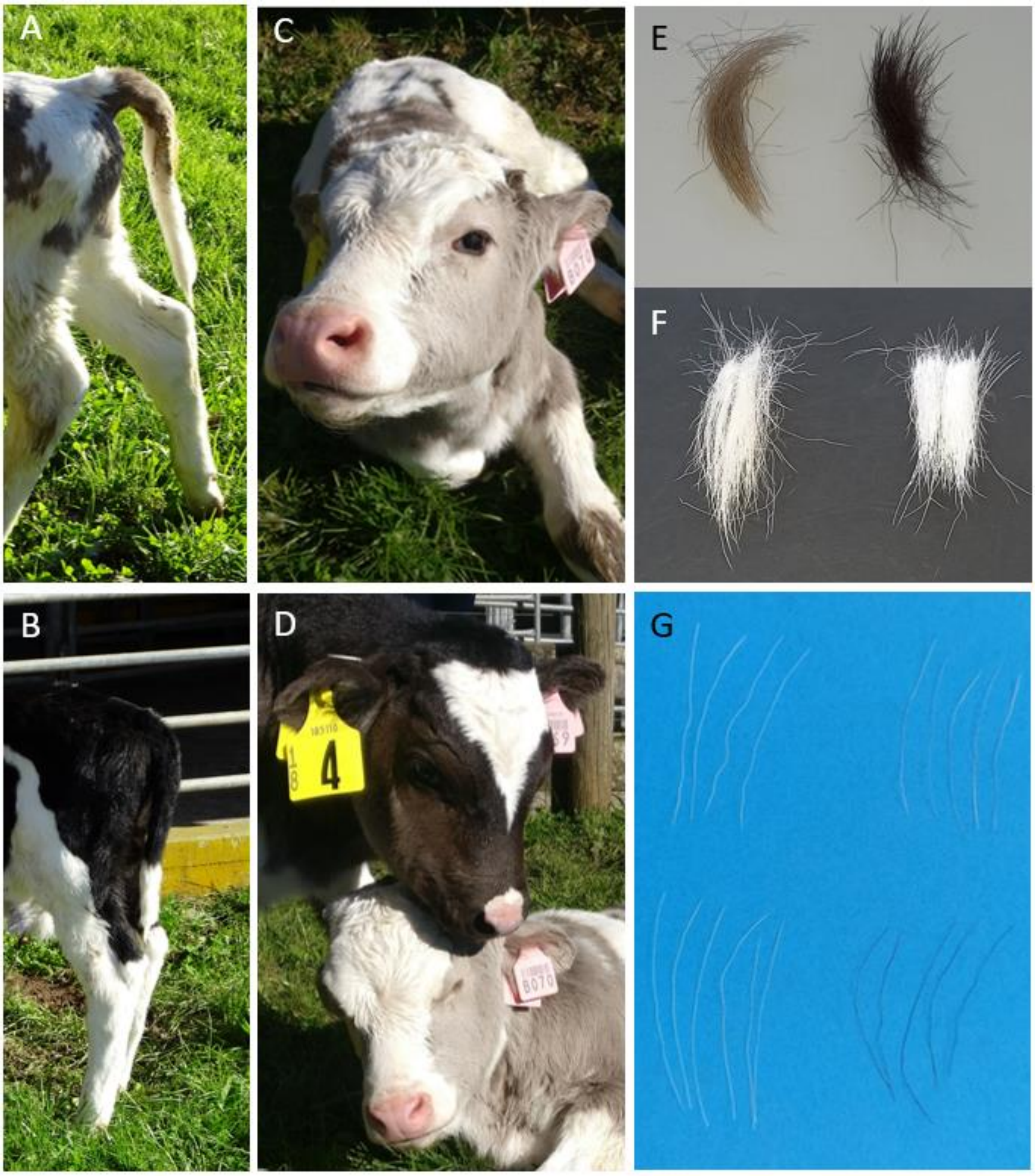

Fig. S6. Absence of characteristic hair phenotypes associated with rat tail syndrome. The tails have a similar appearance with no apparent difference in developing tail switch between edited (A) and control calves (B); C, D) shows the presence of eye lashes and hair in the ears of edited and control calves; E, F, G) the difference in hair length between pigmented and non-pigmented areas is similar in edited and control calves. E: pigmented hair, edited left, control right; F: non-pigmented hair, edited left, control right; G: top (edited); left non-pigmented, right pigmented; bottom (control); left non-pigmented, right pigmented.
